# Predictors of Satisfaction with Autism Treatment Services During COVID-19

**DOI:** 10.1007/s10803-021-05232-0

**Published:** 2021-08-27

**Authors:** Emily F. Ferguson, Maria Jimenez-Muñoz, Harrison Feerst, Ty W. Vernon

**Affiliations:** Koegel Autism Center, University of California Santa Barbara, Santa Barbara, CA 93117 USA

**Keywords:** Autism spectrum disorder, Service satisfaction, Caregivers, COVID-19, Emotion regulation, Telehealth

## Abstract

The COVID-19 pandemic has created unprecedented challenges and disruptions for autistic individuals receiving specialized treatment services. This caregiver-report survey study (*n* = 339) explored predictors of satisfaction with autism services during COVID-19 to improve perceived support for these families. Specifically, we investigated whether service delivery medium (telehealth vs. in person), child’s emotional functioning, and caregiver stress would predict satisfaction with the most highly utilized services. Satisfaction ratings for ABA/behavioral, speech/language, and occupational therapy were lower when delivered via telehealth as compared to in person. Caregivers who reported higher emotional dysregulation in their children were less satisfied with behavioral therapy services. These results provide a critical caregiver-informed perspective on factors influencing satisfaction with specialized autism services during COVID-19.

## Predictors of Satisfaction with Autism Treatment Services During COVID-19

The COVID-19 pandemic has dramatically impacted service provision for autistic individuals and their families (Ameis et al., 2020; Pellicano & Stears, 2020), yet there is limited knowledge about parent satisfaction with these treatment services during COVID-19. One survey 2020 revealed that a large majority of parents of autistic individuals consider the COVID-19 restrictions to be extremely difficult, requiring a substantially higher level of personal commitment to fulfill their child’s needs (Colizzi et al., 2020). Caregiver-report data collected during the pandemic can shed light on the factors that influence service satisfaction levels in order to improve perceived quality of the support for these families. Thus, it is important to seek out caregiver input to meet the diverse service needs of autism families both during the pandemic and its aftermath (Fitzpatrick et al., 2020).

### Specialized Service Needs and Delivery Medium

The varied needs of individuals on the autism spectrum necessitate a broad range of therapeutic services, spanning ABA/behavioral therapy, speech/language therapy, occupational therapy, psychotherapy, adaptive functioning coaching, vocational training, and social skills support. Consistent intervention delivery is key for maintaining routines, promoting individual growth, and providing support to caregivers (Aishworiya & Kang, 2020). In response to COVID-19, many of these services have been temporarily halted, significantly reduced in intensity, and/or switched to video conferencing and telemedicine platforms in an attempt to ensure continuity of care and ongoing learning. The shift to telehealth highlights the ways that technology can be successfully leveraged to address barriers to service access (Solomon & Soares, 2020). Simultaneously, autistic individuals often require specific accommodations and caregiver support to optimally participate in and benefit from online service provision (Ingersoll & Berger, 2015). Within the home environment, caregivers may not feel equipped to provide the same level of care as a specialized therapist, behaviorist, or educator, yet may feel the pressure to replicate this caliber of support. This may lead to increased stress and distress for autism families during COVID-19 (White et al., 2021a, 2021b). Some caregivers may perceive telehealth as a viable alternative, even if it is not their preferred mode of service delivery (White et al., 2021a, 2021b). Further explorations are needed to assess the link between service delivery medium (telehealth vs. in person) and caregiver satisfaction with specific autism therapies and services.

### Emotional and Behavioral Functioning Concerns

Deviations in established routines can contribute to a number of emotional and behavioral challenges for children and adults on the spectrum (Nonweiler et al., 2020; Osborne & Reed, 2009). In the early stages of COVID-19 stay-at-home orders, parents of autistic children reported higher anxiety, meltdowns, and aggressive behaviors (COVID-19 and its Impact on the SPARK ASD Community, 2020), along with increased behavior regulation difficulties (especially when these difficulties existed prior to COVID-19; Colizzi et al., 2020). Ameis et al. (2020) predicted that the pandemic would particularly impact autistic individuals who experience emotion dysregulation, which has been implicated as a core feature of mood and anxiety disorders (Mazefsky et al., 2018a, 2018b). Caregivers have reported that managing emotion dysregulation in their children is a key challenge during the pandemic (White et al., 2021a, 2021b). It remains to be determined whether child’s emotional distress and problems with emotion regulation are factors that influence caregiver-reported satisfaction with treatment services during COVID-19.

### Caregiver Stress Levels and Satisfaction with Services

Parents of autistic children consistently report higher levels of stress than parents of typically developing children and children with other developmental disabilities (e.g. Barroso et al., 2018; Hayes & Watson, 2013; Van Steijn et al., 2014). Recent findings suggest that anxiety and stress have increased for caregivers of autistic children during COVID-19 (Wang et al., 2021). A survey completed by over 8000 caregivers revealed that 97% of parents reported feeling stressed or overwhelmed in response to disruptions in their autistic child’s services (COVID-19 and its Impact on the SPARK ASD Community, 2020). Internationally, Turkish parents of autistic children reported higher levels of anxiety and lower feelings of psychological well-being compared to parents of typically developing children during the pandemic (Ersoy et al., 2020). Additionally, greater stress has been reported for caregivers of autistic individuals who were receiving a greater intensity of services pre-COVID-19 (Manning et al., 2020). For children in need of support for emotion regulation difficulties, disruptions in behavior support plans implemented by support staff at school may lead to greater aggression or meltdowns at home (Courtenay & Perera, 2020), leading to additional stress-inducing challenges for caregivers.

Pre-COVID-19 explorations of caregiver-reported satisfaction with autism services have previously identified several barriers to satisfaction, including limited type and frequency of intervention services available (especially in rural areas) and lower satisfaction as child age increases (Crais et al., 2020; McIntyre & Zemantic, 2017). While other investigations found no association between treatment satisfaction and caregiver stress prior to COVID-19 (Rovane et al., 2020), additional research is needed to discern whether caregiver stress is related to satisfaction with specific autism services during the pandemic.

### Current Study

Although caregiver stakeholder perspectives are important to improving care delivery systems, detailed information on child and caregiver characteristics as they relate to satisfaction with services have not been systematically examined. These examinations are critically important to undertake during COVID-19 and throughout ongoing transitions as the format of service delivery continues to shift. The purpose of the current study was to examine patterns of service access in order to investigate predictors of caregiver-reported satisfaction with the most frequently endorsed services. We aimed to investigate whether service delivery medium (telehealth vs. in person), child’s emotional and behavioral functioning, and caregiver stress would predict service satisfaction levels with the most highly utilized services.

## Methods

### Participants

Eligible participants for this cross-sectional study included parents or other legal guardians (aged 18 or older) of children (aged 2 or older) diagnosed with autism spectrum disorder (ASD) who were residing in the same U.S. state as their child. The mean age of autistic individuals, per caregiver report, was 13.4 years (SD 7.8; Range 2–45 years). Overall, 339 caregivers provided informed consent to start the online survey. Of those, 317 completed over 80% of the survey and 308 completed 100% of the survey. Incomplete responses in primary variables of interest were excluded from our analyses. The majority of respondents identified as mothers (92.9%), followed by fathers (5.0%), and other legal guardians (2.1%). The majority of caregivers identified their child as male (76.4%), followed by female (21.8%), transgender (0.3%), and gender variant/gender non-conforming (0.9%). See Table 1 for more participant demographic information, including race/ethnicity, socioeconomic status, intellectual functioning level, and conversational language level.

**Table 1:** Descriptive table

Domain/item	Number/frequency	Percentage	Mean (SD)
Child's age			13.4 (7.8)
Child's gender (n = 335)			
Male	256	76.4	
Female	73	21.8	
Transgender	1	0.3	
Non-conforming	3	0.9	
Child's race/ethnicity (n = 334)			
White	264	79.0	
Black/AA	15	4.5	
Asian	11	3.3	
Native Am	1	0.3	
Other	10	3.0	
Biracial	25	7.5	
Hispanic/latinx	54	16.2	
Child's cognitive level (n = 330)			
High/very high (IQ of 120 +)	30	9.1	
High/average (IQ of 110–119)	43	13.0	
Average (IQ of 90–109)	57	17.3	
Low/average (IQ 80–89)	38	11.5	
Low/borderline (IQ 70–79)	30	9.1	
Mild/moderate intellectual challenges (IQ of 40–69)	36	10.9	
Moderate/severe intellectual challenges (IQ < 40)	44	13.3	
Child's language level (n = 330)			
No words/usually speaks in single words	82	24.8	
Usually speaks in 10 + single words	17	5.2	
Usually speaks in phrases (2–3 words together)	57	17.3	
Usually speaks in full sentences	170	51.5	
Respondent SES bracket (n = 338)			
Less than $20,000	32	9.5	
$20,000 to $39,999	37	10.9	
$40,000 to $59,999	27	8.0	
$60,000 to $79,999	48	14.2	
$80,000 to $99,999	49	14.5	
$100,000 to $139,999	33	9.8	
$140,000 to $179,999	36	10.7	
$180,000 to $199,999	15	4.4	
$200,000 or more	31	9.2	
Respondent's relationship to child (n = 339)			
Mother	315	92.9	
Father	17	5.0	
Other legal guardian	7	2.1	

### Recruitment

Data were collected from caregivers via an online Qualtrics survey that was published from May 27, 2020 to July 10, 2020. The survey was disseminated via targeted social media posts, autism mailing lists/listservs, and professional relationships with other autism centers, organizations, and foundations. Families were recruited from across the United States, with the majority of families from California (22.7%), followed by North Carolina (16.5%), New York (9.4%), Oregon (6.8%), Texas (5.9%), Pennsylvania (4.1%), Florida (3.8%), South Carolina (3.5%), Virginia (3.2%), New Jersey (2.7%), and Wisconsin (2.4%). The remaining 28 states where caregivers responded to the survey were represented by less than 2% of respondents. In an effort to disseminate available resources for the ASD community, participants or those who opted not to complete the survey were provided with a downloadable list of nationwide COVID-19 resources. Participants who opted to share their emails were entered into a drawing to win one of ten $25 online shopping gift cards.

### Survey Content

The caregiver-report survey included demographic information, a standardized measure of caregiver stress, and measures about their child (aged 2 or older) on the autism spectrum. Caregivers completed the online survey in a mean time of 13.9 min.

### Demographic Variables

Caregivers first completed questions about their relationship to the child (mother, father, other legal guardian), U.S. state of residence, socioeconomic status, and health insurance status. Child characteristics were then captured through a set of diagnostic classification questions that provided information on age (continuous variable from 2 years and older), gender, race/ethnicity, language level, and estimated intellectual functioning level (Table 1). They were asked to refer to available developmental, psychological, or school testing results if available.

### Service Access and Delivery Medium during COVID-19

Caregivers were prompted to endorse all services that that their autistic child was receiving during COVID-19 from a list that included: psychotherapy; speech/language therapy, occupational therapy; applied behavior analysis (ABA)/behavioral therapy; social skills training; vocational/job skills training; group home/assisted living program; disability services at a college or university; none; other services (respondent specified); and prefer not to answer. For each endorsed service, caregivers identified whether their child was currently receiving the service in person, via telehealth, or through a hybrid model (combination of telehealth and in person).

### Satisfaction with Services During COVID-19

For each service that caregivers endorsed their autistic child was receiving during COVID-19, they were prompted to respond to the following statement: “My general feeling about this service’s ability to respond to my child’s needs *during* COVID-19 is: __.” Satisfaction with the service was rated on a 5-point scale (*1* = I am very much dissatisfied, *2* = I am somewhat dissatisfied, *3* = I feel neutral, *4* = I am somewhat satisfied, *5* = I am very much satisfied). This question was adapted from the Therapy Attitude Inventory (TAI; Eyberg, 1993), which has been shown to have adequate reliability, validity, and sensitivity to treatment satisfaction (Brestan et al., 1999). Service satisfaction scores were collected for each individual service.

### Child and Caregiver Predictor Variables

*Emotion Regulation* The Emotion Dysregulation Inventory (EDI; Mazefsky et al., 2018a, 2018b, 2020) is an informant report measure of emotion dysregulation, or emotional distress and problems with emotion regulation that is currently normed for individuals aged 6 years and older. Caregivers rated items on a five-point scale based on their child’s behavior over the past 7 days from “*0* = not at all” to “*4* = very severe.” The EDI yields separate scores for *Reactivity*, or rapidly escalating, intense, and poorly regulated emotional responses (characterized by anger/irritability) and for *Dysphoria*, or sadness, unease, low motivation, and anhedonia. The short form of the EDI includes 13-items total, with 7 items for Reactivity and 6 items for Dysphoria. The EDI has been found to have good reliability in samples of individuals across the spectrum of ASD (α = 0.94; Conner et al., 2018). EDI raw scores for Reactivity and Dysphoria were converted to *t*-scores using a clinical (ASD) normative sample (Mazefsky et al., 2018a, 2018b). The mean reactivity *t*-score (*n* = 318) was 49.6 (SD 8.6) and the mean dysphoria *t*-score was 51.8 (SD 8.6). For reactivity, t-scores above 46.9 are considered clinically elevated, whereas t-scores above 52.2 can be considered clinically elevated for dysphoria in the ASD normative sample.

*Caregiver Global Stress Level* Caregiver stress levels were measured with the Perceived Stress Scale (PSS; Cohen et al., 1994). This is a brief 10-item self-report questionnaire that assesses perceptions of global stress during the last month. The PSS has been shown to have good concurrent validity (α = 0.90) in recent studies involving caregivers of children with ASD (Lovell & Wetherell, 2015). Respondents were asked to indicate how often they felt or thought in a certain way on a scale ranging from “0 = never” to “4 = very often”. Individual scores on the PSS can range from 0 to 40 with higher scores indicating higher perceived stress. Scores ranging from 0 to 13 are considered “low stress”, those from 14 to 26 “moderate stress,” and scores in the 27 to 40 range are deemed “high” stress. The mean PSS score (*n* = 309) was 21.8 (SD 3.7), indicating that caregivers rated their stress levels in the moderate range on average.

### Data Analysis

We first examined caregiver-reported service utilization to select the most highly accessed services for regression analyses. Next, we ran Pearson’s correlations (*r*) to determine associations between variables and to rule out multicollinearity (Mansfield & Helms, 1982). Sociodemographic and child cognitive variables were not significantly correlated with satisfaction with services and were thus excluded from the following regression models. Given the age range of autistic individuals in our sample, age was included as a covariate in the following regression models.

Standard multiple linear regression models examined satisfaction with ABA/behavioral therapy services, speech/language therapy, psychotherapy, and occupational therapy (as the most highly utilized services). EDI reactivity *t*-scores, EDI dysphoria *t*-scores, caregiver stress, service delivery medium, and child’s age were added simultaneously into these models to assess predictors of caregiver-reported satisfaction with autism support services during COVID-19. Beta coefficients were used to determine the magnitude of prediction for each independent variable.

## Results

### Patterns of Service Access and Satisfaction During COVID-19

Of the respondents, 317 caregivers endorsed the types of services that their autistic children were receiving during COVID-19 and satisfaction with those services (Table 2). In the midst of the pandemic, 35.33% of respondents noted that their autistic child was not currently receiving any services. Of the families who were receiving services, speech/language therapy was the most commonly endorsed service, with 29.34% of participants reporting receiving this type of service. This was followed by ABA/behavioral therapy (26.47%), psychotherapy (18.93%), and occupational therapy services (18.93%). The least commonly endorsed services focused on the needs of older autistic individuals, which was anticipated given the mean age of the respondents’ children. These services included including vocational/job skills (3.79%), group home/assisted living (2.20%), and disability services at a university (0.95%).

**Table 2:** Mean satisfaction with services

Type of service	n	Satisfaction with service, *M*	Telehealth, *n*	Telehealth satisfaction, *M*	In person, *n*	In person satisfaction, *M*	Hybrid, *n*	Hybrid satisfaction, *M*
Psychotherapy	60	3.27	43	3.28	7	4	10	2.7
Speech/language	93	3.24	60	2.93	18	3.94	15	3.6
ABA/behavioral therapy	84	3.35	25	3.20	46	4.17	13	2.39
Occupational therapy	60	3.1	34	2.68	19	3.79	7	3.29
Social skills	36	2.92	22	2.77	7	3.29	7	3.00
Vocational	12	3.25	8	3.13	3	4.33	1	3.25
Group home/assisted living	7	3.29	0	N/A	5	2.8	2	3.28
Disability services at University	3	1.33	3	1.33	0	N/A	0	N/A
Other	47	3.19	22	3.09	19	3.42	6	2.83
None	112	N/A	N/A	N/A	N/A	N/A	N/A	N/A

An examination of caregiver satisfaction by service type indicates that, on average, caregivers were most satisfied with ABA/behavioral therapy during the pandemic (*M* = 3.7, SD 1.36). Satisfaction ratings for psychotherapy (*M* = 3.27, SD 1.40), speech/language (*M* = 3.24, SD 1.3), occupational therapy (*M* = 3.10, SD 1.45), social skills training (*M* = 2.92, SD 1.16), vocational services (*M* = 3.25, SD 1.22), and services provided in group homes/assisted living contexts (*M* = 3.29, SD 1.38) all indicated average/neutral feelings about these services during COVID-19. The three respondents who reported on satisfaction with disability services at a college/university noted high levels of dissatisfaction (*M* = 1.33, SD 0.58).

Telehealth was the most common medium of delivery for psychotherapy (71.67%), speech/language (64.5%), occupational therapy (56.67%), social skills services (61.11%), vocational interventions (66.67%), and disability services provided by colleges (100%). In contrast, the majority of respondents indicated that ABA/behavioral therapy (54.76%) and group home/assisted living services (71.43%) were delivered in person. Mean satisfaction ratings for each of these service delivery mediums are provided in Table 2.

### Predictors of Caregiver-Reported Satisfaction with Services

*ABA/Behavioral Therapy* The first linear regression model examined satisfaction with ABA/behavioral therapy services. Child’s reactivity, dysphoria, age, caregiver stress, and service delivery medium accounted for 30.9% of the variance in behavioral service satisfaction ratings (*R*^*2*^ = 30.9; *F*_6,72_ = 5.37, *p* < .001). Caregivers reported significantly lower satisfaction for behavioral services delivered via telehealth (*B* =  − .301, *t* =  − 2.87, *p* = .005) as compared to services delivered in person. Higher child dysphoria significantly predicted lower behavioral therapy satisfaction (*B* =  − .312, *t* =  − 2.57, *p* = .012). A summary of regression coefficients for each of the predictor variables is provided in Table 3.

**Table 3:** Regression analysis for caregiver-reported service satisfaction with ABA/other behavioral services

Variable	Unstandardized coefficients		Standardized coefficients			95.0% confidence interval for B	
	*B*	SE B	β	t	*p*	Lower bound	Upper bound
Intercept	4.040**	.267	–	15.129	.000	3.507	4.572
Child's age	.005	.021	.076	.614	.800	− .037	.048
EDI reactivity	.014	.023	− .147	.614	.541	− .032	.060
EDI dysphoria	− .060	.023	− .312	− 2.567	.012	− .106	− .013
Parent stress	− .005	.039	− .015	− .141	.888	− .083	.072
Telehealth medium	− .893**	.311	− .301	− 2.871	.005	− 1.513	− .273
Hybrid medium	− 1.633**	.407	− .438	− 4.082	.000	− 2.475	− .851
${R}^{2}$	.309						
${Adjusted\,R}^{2}$	.252						
*F*	5.370**						

*EDI* emotion dysregulation inventory t-score for autistic individuals, *Parent Stress* parent endorsements on the Perceived Stress Scale, *Hybrid Medium* combination of in-person and telehealth service delivery

*p < .05 (two-tailed test), **p < .01 (two-tailed test)

*Speech/Language Therapy* The next linear regression model examined satisfaction with speech/language services. Child’s reactivity, dysphoria, age, caregiver stress and service delivery medium accounted for 28.8% of the variance in speech satisfaction ratings (*R*^*2*^ = 28.8; *F*_6,83_ = 5.59, *p* < .001). Higher child’s age predicted higher caregiver satisfaction with speech services (*B* = .210, *t* = 2.17, *p* = .033). In addition, caregivers reported significantly lower satisfaction for speech services delivered via telehealth (*B* =  − .444, *t* =  − 3.68, *p* < .001) as compared to services delivered in person. A summary of regression coefficients for each of the predictor variables is provided in Table 4.

**Table 4:** Regression analysis for caregiver-reported service satisfaction with speech services

Variable	Unstandardized coefficients		Standardized coefficients			95.0% confidence interval for B	
	*B*	SE B	β	t	*p*	Lower bound	Upper bound
Intercept	3.485**	.369	–	9.450	.000	2.751	4.218
Child's age	.057	.026	.210	2.171	.033	.005	.110
EDI reactivity	− .027	.022	− .166	− 1.196	.235	− .072	.018
EDI dysphoria	− .047	.024	− .265	− 1.988	.050	− .094	.000
Parent stress	.024	.036	.066	.669	.505	− .047	.095
Telehealth medium	− 1.276**	.347	− .444	− 3.678	.000	− 1.966	− .586
Hybrid medium	− .481	.436	− .131	− 1.105	.273	− 1.348	.385
${R}^{2}$	.288						
${Adjusted \,R}^{2}$	.236						
*F*	5.593**						

*EDI* emotion dysregulation inventory t-score for autistic individuals, *Parent Stress* parent endorsements on the Perceived Stress Scale, *Hybrid Medium* combination of in-person and telehealth service delivery

*p < .05 (two-tailed test), **p < .01 (two-tailed test)

*Psychotherapy* The linear regression model examining satisfaction with psychotherapy services showed that child’s reactivity, dysphoria, age, caregiver stress and service delivery medium accounted for 30.0% of the variance in psychotherapy satisfaction ratings (*R*^*2*^ = 29.0; *F*_6,51_ = 3.64, *p* = .004). Caregivers did not report significantly lower satisfaction for psychotherapy delivered via telehealth as compared to services delivered in person (*B* =  − .716, *t* =  − 1.29, *p* = .204). Higher caregiver stress predicted higher satisfaction with psychotherapy (*B* = .278, *t* = 2.33, *p* = .024). A summary of regression coefficients for each of the predictor variables is provided in Table 5.

**Table 5:** Regression analysis for caregiver-reported service satisfaction with therapy/counseling services

Variable	Unstandardized coefficients		Standardized coefficients			95.0% confidence interval for B	
	*B*	SE B	β	t	*p*	Lower bound	Upper bound
Intercept	3.804**	.602	–	6.320	.000	2.596	5.012
Child's age	.033	.030	.134	1.100	.276	− .027	.092
EDI reactivity	− .050	.028	− .232	− 1.769	.083	− .106	.007
EDI dysphoria	− .032	.024	− .170	− 1.333	.189	− .079	.016
Parent stress	.134*	.058	.278	2.332	.024	.019	.250
Telehealth medium	− .716	.557	− .234	− 1.286	.204	− 1.833	.402
Hybrid medium	− 1.422*	.651	− .392	− 2.184	.034	− 2.729	− .115
${R}^{2}$	.300						
${Adjusted\, R}^{2}$	.217						
*F*	3.635**						

*EDI* emotion dysregulation inventory t-score for autistic individuals, *Parent Stress* parent endorsements on the Perceived Stress Scale, *Hybrid Medium* combination of in-person and telehealth service delivery

*p < .05 (two-tailed test), **p < .01 (two-tailed test)

*Occupational Therapy* Lastly, a linear regression model examined satisfaction with occupational therapy services. Child’s reactivity, dysphoria, age, caregiver stress and service delivery medium accounted for 30.2% of the variance in occupational therapy satisfaction ratings (*R*^*2*^ = 30.2; *F*_6,52_ = 3.75, *p* = .004). Higher parental stress predicted higher satisfaction with occupational therapy services (*B* = .271, *t* = 2.09, *p* = .042). In addition, caregivers reported significantly lower satisfaction for occupational therapy services delivered via telehealth (*B* = − .336, *t* = − 2.53, *p* = .015) as compared to services delivered in person. A summary of regression coefficients for each of the predictor variables is provided in Table 6.

**Table 6:** Regression analysis for caregiver-reported service satisfaction with occupational therapy services

Variable	Unstandardized coefficients		Standardized coefficients			95.0% confidence interval for B	
	*B*	SE B	β	t	*p*	Lower bound	Upper bound
Intercept	3.140**	.432	–	7.275	.000	2.274	4.006
Child's age	.058	.035	.196	1.662	.103	− .012	.129
EDI reactivity	− .039	.029	− .214	− 1.348	.184	− .096	.019
EDI dysphoria	− .050	.031	− .266	− 1.628	.110	− .111	.012
Parent stress	.105	.051	.271	2.085	.042	.004	.207
Telehealth medium	− .974	.386	− .336	− 2.526	.015	− 1.749	.200
Hybrid medium	− .421	.568	− .095	− .741	.462	− 1.561	− .720
${R}^{2}$	.302						
${Adjusted\,R}^{2}$	.222						
*F*	3.751**						

*EDI* emotion dysregulation inventory t-score for autistic individuals, *Parent Stress* parent endorsements on the Perceived Stress Scale, *Hybrid Medium* combination of in-person and telehealth service delivery

*p < .05 (two-tailed test), **p < .01 (two-tailed test)

## Discussion

This caregiver-report survey study explored predictors of satisfaction with services for autistic individuals (aged 2 and older) during the COVID-19 pandemic. Specifically, this study investigated the impact of service delivery medium, child’s emotional and behavioral functioning, and caregiver stress levels on satisfaction with autism support services. Our findings revealed that caregiver-reported satisfaction was lower for ABA/behavioral, speech/language, and occupational therapy services delivered via telehealth than for those delivered in person. Caregivers who endorsed higher emotional dysregulation for their autistic children reported lower satisfaction with ABA/behavioral therapy services. These findings lay the foundation for further explorations of factors that drive caregiver stakeholder satisfaction with autism services to guide targeted improvement efforts.

### Patterns of Service Access and Satisfaction During COVID-19

Initially, we characterized patterns of service access and satisfaction as reported by caregivers of autistic individuals across the U.S. Notably, 35.33% of caregivers reported that their autistic children and adults were not receiving any autism support services during the pandemic. These findings are consistent with a large-scale SPARK survey from March 2020, in which only 35% of families reported receiving remote services and 63% of families reported severe disruptions in services during the pandemic (COVID-19 and its Impact on the SPARK ASD Community, 2020). In our study, among those who reported access to services, speech/language therapy was the most highly endorsed service, followed by ABA/behavioral therapy, psychotherapy, and occupational therapy services.

Mean satisfaction scores for individual services during COVID-19 revealed that ABA/behavioral therapy had the highest satisfaction ratings across all delivery mediums (albeit with variability in responses), whereas the handful of satisfaction ratings on virtual disability services at a college/university yielded consistently low ratings (*n* = 3). For all other services, satisfaction scores were generally neutral, which may suggest that caregivers are not fully satisfied with these services. While additional respondents of college-aged children are needed for more definitive claims, these low ratings align with calls to enrich supports for older autistic individuals during the pandemic and more broadly (e.g. Bishop-Fitzpatrick & Kind, 2017; Howlin & Moss, 2012).

### Predictors of Caregiver-Reported Satisfaction with Services

To better understand factors that predict satisfaction with services, we explored associations between emotion regulation, stress levels, and service delivery medium on satisfaction with the most frequently endorsed autism support services.

*Child Emotion Regulation* Emotion regulation for autistic individuals was captured via the tendency to have intense or extreme emotional reactions (EDI reactivity score) and emotional unease or sadness (EDI dysphoria score). Notably, higher dysphoria significantly predicted lower caregiver-reported satisfaction with ABA/behavioral therapy services. Given that dysphoria often underlies psychiatric disorders such as anxiety or depression, this finding suggests that greater mental health concerns for children with ASD may contribute to diminished parental satisfaction with certain autism support services (Maddox et al., 2020). Specifically, it may be important to incorporate supports for mental and emotional health into ABA/behavioral services for autistic individuals (Aishworiya & Kang, 2020; Fong et al., 2020; Parker et al., 2020).

In contrast, higher child dysphoria scores did not predict decreased satisfaction with speech/language services, therapy, or occupational therapy services. This suggests that parental expectations for the type of care provided by ABA/behavioral services may differ. ABA/behavioral services are focused on goals related to child’s conduct, which can be greatly affected by the lower mood and emotional unease encompassed by the dysphoria score (Gore & Baker, 2017). On the other hand, speech/language and occupational therapy services target a specific goal, namely language development and motor skills, which might not be impacted by mental health concerns to the same degree. It is also possible that caregivers have to play a greater role engaging children who are demonstrating sadness or unease via telehealth ABA/behavioral therapy sessions, leading to decreased satisfaction with behavioral services in this format.

*Caregiver Stress* Higher parental stress was a significant predictor of higher satisfaction with both psychotherapy and occupational therapy services. While more research with larger samples is needed to replicate and expand upon these findings, the associations between parental stress and service satisfaction for two services may indicate that parental stress is an important variable to consider when evaluating satisfaction with services. Psychotherapy services for autistic children may play a role in enhancing feelings of support for the family as a whole, which may be especially impactful for caregivers who experience high stress levels.

*Service Delivery Medium* The period of COVID-19 and social distancing restrictions has illustrated ways that telehealth may be leveraged to address barriers to service access (Solomon & Soares, 2020). As more providers adopt this medium during the pandemic and beyond, it is important to consider caregiver satisfaction with this model. Our findings suggest that parental satisfaction was lower for ABA/behavioral services, speech/language, and occupational therapy services delivered through an online, telehealth medium. These findings are consistent with other caregiver reports that the benefits of services delivered via telehealth are diminished for all service types during COVID-19 (White et al., 2021a, 2021b). Service providers have previously expressed concerns about telehealth disrupting the provider–patient relationship (Committee on Pediatric Workforce, 2015), which could in turn contribute to higher dissatisfaction. Furthermore, providers may experience discomfort with unfamiliar technologies that interferes with effective service delivery (Utidjian & Abramson, 2016). However, the telehealth-only medium did not significantly predict lower satisfaction with psychotherapy services, which may suggest that the face-to-face psychotherapy format more readily adapted to online delivery. With the potential for ongoing pandemic-related transitions that disrupt service provision, it would be beneficial to continue to explore the merits of hybrid service delivery models that combine in person and telehealth elements (White et al., 2021a, 2021b).

### Limitations

The current research provides an important perspective on factors influencing caregiver-reported satisfaction with specialized autism services. However, some study limitations are important to consider. First, the data presented here are cross-sectional. As a result, analyses can only identify associations; they cannot determine causality. A complex array of caregiver demands during COVID-19 (e.g., number of children at home, financial stress, illness) and other unmeasured variables likely contributed to variance in service satisfaction ratings during COVID-19 (e.g. Baweja et al., 2021; Vasudevan et al., 2021). Second, an inherent limitation of an online survey study is that recruitment is limited to individuals who have access to a cell phone or computer and a stable internet connection, which may limit representation of caregivers from lower SES families. Given the sharp rise in unemployment during COVID-19, it will be important to assess needs and to direct service delivery efforts to autism families who may have fallen through the cracks (Pellicano & Stears, 2020). Finally, service satisfaction data is limited to quantitative responses, which do not provide a comprehensive understanding of caregivers’ opinions. Future efforts will involve both quantitative (e.g. Gerber et al., 2016) and qualitative (e.g. Crais et al., 2020) research methods to capture the range of family factors that drive service satisfaction ratings.

Despite the consistent prevalence of ASD across ethnic and racial groups (Centers for Disease Control and Prevention, 2014), minority groups continue to be underrepresented in autism research (Harris et al., 2020). The current survey study is no exception, with the majority of respondents identifying as White/Caucasian. In an effort to understand treatment satisfaction for historically underserved populations (Broder-Fingert et al., 2020; Flores et al., 2002), future research will focus on targeted recruitment efforts to investigate treatment access and satisfaction for racial/ethnic minority groups. Finally, we aim to expand recruitment efforts to reach more caregivers of individuals with profound autism, a population that is critically underrepresented in the research literature (Siegel, 2018), and in need of targeted service efforts during COVID-19 and beyond (Brondino et al., 2020).

### Future Directions

The findings presented suggest that greater emotional distress for autistic individuals contributes to lower caregiver service satisfaction ratings with ABA/behavioral services. Thus, introducing additional emotion regulation support might have a positive impact on caregivers’ satisfaction with ABA/behavioral therapy and reduce stress for the family as a whole. Given that improvements in emotion regulation can also lead to reductions in anxiety, depression, and problem behaviors (White et al., 2018), it is important that services target positive coping strategies to enhance emotion regulation skills and psychological wellbeing.

Existing literature provides examples of treatment programs designed to enhance emotion regulation skills in autistic individuals. For example, a manualized intervention that adapts Cognitive-Behavioral Therapy to specifically target emotion regulation skills leads to improvements in internalizing and externalizing symptoms, adaptive skills, and anxiety for children on the autism spectrum (Weiss et al., 2018). Similarly, the EASE program improves emotion regulation skills for verbal autistic adolescents and adults through the use of mindfulness and distress tolerance strategies (Conner et al., 2018). Components of these programs that teach autistic individuals how to recognize and respond to emotions could be incorporated into services and therapies for children exhibiting greater emotional distress. The next step in this line of research is to explore the added value of emotion regulation skills training on both client and caregiver satisfaction with behavioral therapies.

Finally, our findings suggest that caregiver satisfaction is lower for ABA/behavioral, speech/language, and occupational therapy services delivered via telehealth as compared to in person. However, initial findings from a sample of 17 cases that transitioned from in person to telehealth delivery during COVID-19 found that the majority of autistic clients continued to benefit from these services in reaching their behavioral goals (Pollard et al., 2021). Parental involvement in services delivered via telehealth may also positively impact family functioning as a whole (Factor et al., 2019). In spite of these promising findings, our results suggest that more work is needed to understand variability in satisfaction ratings for telehealth services to increase caregivers’ satisfaction with this delivery medium. Clinically, offering additional appointments to clarify autistic individuals’ preferences for using telehealth may enhance satisfaction and engagement (Spain et al., 2021). Development of good practice guidelines for the delivery of specific services via telehealth may also prove very useful (Spain et al., 2021). Future research should gather qualitative data that allows families to express their unique experiences and satisfaction with services delivered via telehealth, by service type, with the goal of developing guidelines for service providers.

### Conclusion

In spite of the catastrophic impacts of COVID-19, the pandemic also provides an opportunity to increase satisfaction with services for autistic individuals and their families (Ameis et al., 2020). The results of this survey are an important first step in addressing a critical lack of knowledge nationwide about predictors of caregiver-reported satisfaction with autism therapies and services. Ultimately, it is our hope that these survey findings will help to inform next steps in gathering stakeholder perspectives to improve satisfaction with autism treatment services.
